# On the Influence of Parasites in the Production of Disease

**Published:** 1853-09

**Authors:** Joseph Leidy

**Affiliations:** Professor of Anatomy in the University of Pennsylvania


From the Virginia Medical and Surgical Journal.

*On the Influence of Parasites in the Production of Disease.* By JOSEPH LEIDY, M. D., Professor of Anatomy in the University of Pennsylvania.

IN many animals entozoa and entophyta are almost never absent, and probably when in their natural habitation, and few in number, or not of excessive size, are harmless, as observed by Dujardin in the introduction of his excellent work on Intestinal Worms:—"Les helminthes se developpent dans un site qui leur convient, sans nuire plus que les lichens sur l'ecorce d'un arbre vigoureux. Ils ne peuvent devinir nuisibles, generalement, que par suite d'une multiplication excessive, laquella semble alors etre une des consequences d'un affaiblissement provenant d'une tout autre cause, d'une mauvaise alimentation, du sejour dans un lieu froid et humide, etc.: sans cela, les helminthes naissent et meurent dans le corps de leurs hotes, et peuvent paraitre et disparaitre alternativement sans inconvenients."

Many important diseases have been supposed to originate from parasitic animals and vegetables. The former are not the true entozoa, for these are too large, and may be detected by the naked eye, but they are considered to be animalculæ so small that they cannot be discovered even with the highest powers of the microscope. But, independent of the fact that the existence of such entities is a mere supposition, none of the well known animalculæ

are poisonous. At various times I have purposely swallowed large draughts of water containing myriads of *Monas*, *Vibrio*, *Euglenia*, *Volvox*, *Leucophrys*, *Paramecium*, *Vorticelli*, etc., without ever having perceived any subsequent effect.

The production of certain diseases, however, through the agency of entophyta, is no longer a subject of doubt; as in the case of Muscardine in the Silk-worm, the Mycodem of Porrigo favosa in Man, etc.; but that malarial and epidemic fevers have their origin in cryptogamic vegetables or spores requires a single proof.* If such were the case minute vegetable or spores conveyed through the air, and introduced into the body in respiration, could be detected. The minutest of all known living beings is the *Vibrio lineola* of Muller, measuring only the 36,000 of an inch, and the smallest known vegetable spore is very much larger than this, whilst particles of inorganic matter can be distinguished the 200,000 of an inch in size.

I have frequently examined the rains and dews of localities in which intermittents were epidemic upon the Schuylkill and Susquehanna rivers, but without being able to detect animalculæ, spores, or even any solid particles whatever. I have examined the air itself for such bodies, by passing a current through clear water. This was done by means of a bottle, with two tubes passing through a cork stopper; one tube dipping into the water, the other reaching not quite to its surface. By sucking upon the latter tube, a current of air passed through the former, and was deprived in its course of any solid particle. Ordinarily, when the atmosphere was still, early in the morning, or in the evening, neither spores nor animalcules could be detected. When piles of decayed sticks or dry leaves were stirred up, or the dust was blown about by the wind, a host of most incongruous objects could be obtained from the air; none, however, which could be supposed capable of producing diseases.

To assert, under these circumstances, that there are spores and animalculæ capable of giving rise to epidemics, but not discernable by any means at our command, is absurd; as it is only saying in other words that such spores and animalculæ are liquid and dissolved in the air, or in a condition of chemical solution. That the air may be poisoned by matters incapable of detection by the chemist, is proved by the emanations from such plants as the *Rhus vernix*, *Hippomane mancinella*, etc.—*Smithsonian Contributions to Knowledge.*

---

*See an ingenious little work by my distinguished friend, Dr. J. K. Mitchell, "On the Cryptogamus Origin of Malarious and Epidemic Fevers."

